# A peripatetic pediatrician's journey into pediatric rheumatology: Part II

**DOI:** 10.1186/1546-0096-5-14

**Published:** 2007-06-21

**Authors:** Earl J Brewer

## Abstract

Earl Brewer discusses his journey into pediatric rheumatology from 1958 to retirement in 1990 in three parts.

## Part II: The PRCSG and the Park City meetings in the US

### IV Pediatric Rheumatology Collaborative Study Group (PRSCG) – 1973

The beginning of the PRSCG was part of the evolving plan for the future of pediatric rheumatology in the US. I was impressed with the work of Maxwell Finland in Boston in an earlier era when he cobbled together a group of clinicians to effectively study antibiotics. I knew that we had to develop methods to study medicines for children with arthritis in the US. We were trapped in a circular equation. There were many drugs coming to market for adults with arthritis. I believed that many of these new drugs were non-specific for pain relief and would be used to help all types of pain as well as inflammation. Studies were performed in adults with arthritis. No studies were being performed in children because there were thought to be too few children with arthritis. Therefore the pharmaceutical companies could not justify studies in children for economic reasons. Therefore we had no approved drugs to give arthritic children because the efficacy and safety were not studied in children. It was interesting to me to learn that aspirin was grandfathered in as an approved drug when the US Federal Drug Administration (FDA) was first created many years ago.

I had previously studied indomethocin and acetaminophen in a blinded study of acute control of high fever in children. I conducted the study at the Ben Taub Hospital in Houston. It was published in Arthritis and Rheumatism in 1968. The study was able to show that indomethocin was superior to acetaminophen in controlling fever. This was a short usage study, but no adverse reactions occurred in the short dosage regimen. This study, however, did not address the problem of obtaining approval for the use of indomethacin in children.

Dr. James Gleichart of Abbott Laboratories contacted me concerning an anti-inflammatory drug and a possible study. This was the first interest of a drug company in studying arthritis drugs in children. He informed me that they were two years from studies in adults or children. Nothing came of the contact.

The beginning of the remarkable era of the PRCSG occurred when Dr. Stanley Gottlieb of McNeil Laboratories expressed interest in children's studies for a new drug, tolmetin, a nonsteroidal anti-inflammatory agent. Ralph Wedgwood, a pioneering pediatric immunologist and rheumatologist at the University of Washington in Seattle, and I met with Stan Gottlieb in San Francisco. Ralph trained at the Children's Hospital in Boston several years before I was there. We both had a great interest in children with arthritis and knew each other.

The meeting was an amazing watershed. McNeill was prepared to study tolmetin in children effectively. They already were leaders in medicines for children with such drugs as acetaminophen. They had moved into the new field of NSAIDS and were receptive to helping children. I believe that Dr. John Harter of the approval group at the FDA also encouraged them to be in contact. Ralph, a member of the Wedgwood China family in England, was a bubbly, gregarious, outspoken Englishman and had bolted for the colonies and trained in Boston. Ralph was interested in helping with the effort to develop a way to study these drugs but was not interested in doing the studies himself. We developed with Stan Gottlieb what became Segment I, Segment II, and Segment III Tolmetin studies.

**Figure 1:**
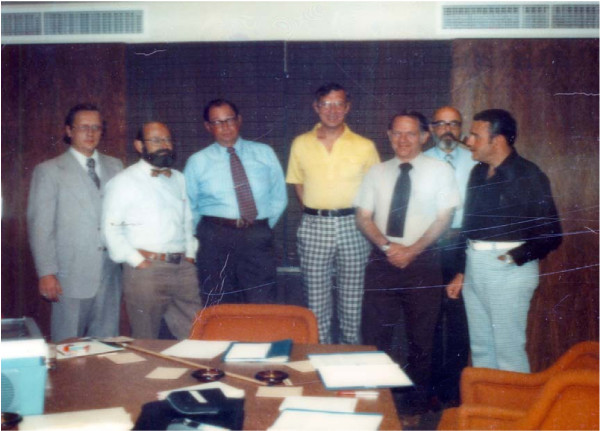
PRCSG Group 1975. From left Drs. Adamski [McNeil Lab], John Baum, Chester Fink, Earl Brewer, Virgil Hanson, Joseph Levinson, & Stanley Gottlieb [McNeil Lab]. Dr. Jane Schaller is not pictured.

After this pivotal meeting, our already collegial group studying criteria entered into active plans to develop our methodology for drug studies and formed what became known as the Pediatric Rheumatology Collaborative Study Group, the PRCSG. The first members were Earl Brewer, chair, Houston; John Baum, Rochester; Virgil Hanson, Los Angeles, Chester Fink, Dallas; Jerry Jacobs, New York City; Joseph Levinson, Cincinnati; Jane Schaller, Seattle. [Figure 1] We had spirited discussions on many occasions regarding the structure of the open, double-blinded, and follow-up studies. We developed the forms and methodology of study with McNeil Laboratories, Dr. John Harter of the FDA, and Dr. Stan Gottlieb.

I am moving ahead of the story here, but we later had a terrible disappointment after finishing the first landmark studies. All of our studies were omitted from McNeil's massive Excerpta Medica publication of the major studies of tolmetin done by Dr. John Ward of the University of Utah and his cooperative adult group sponsored by the NIH. Someone in the marketing department had talked to someone on Wall Street who said that some of the analysts were concerned that a drug that was effective and safe in children must be too weak for adults in comparison with other drugs. I still have the letter from McNeil telling me of their decision not to publish. I was really distressed. Distressed can be spelled in several ways. Fortunately, the FDA approved Tolmetin for usage in children later. We, of course, published the work in the Journal of Pediatrics in 1977 [1]. The publication was a new experience for all of us. A big question was not the data, but how should we list names on the publication and in what order. My main interest was in putting in place a collegial way of listing the first author. Even though I had put the whole arrangement together and was the coordinating center, I felt that we should alternate who would be the first author. It was not the brightest decision, but it solved a problem and preserved our collegial spirit at the time.

Another landmark event occurred in 1976. Edward H. Giannini, a graduate student at the University of Texas School of Public Health, was also employed at the Methodist Hospital and Baylor. He worked with the new NIH CLINFO computer program that effectively became the Internet. I employed Ed to help with our data. Of course, the rest is history. He completed his Doctor of Public Health in 1979, and became our fulltime senior scientist of the PRCSG. Ed was and is gifted in data, epidemiology, and statistics. He and I became instant and close friends. He and his colleagues took PRCSG, which continues today, to several new levels.

## Philosophy of Segment I, II, AND III Drug Studies

It is useful to relate a little of the philosophy of our studies. For detailed descriptions of the design and methodology, the reader is directed to Journal of Rheumatology 9:1, 1982. The purpose of our studies was to study anti-rheumatic and anti-inflammatory drugs for efficacy, safety, and dosage already studied in adults. The NSAIDs and SAARDs were studied identically. The only variables that differed were duration of the studies and criteria for entry. Our concept of study was to perform exploratory, open label, short-term studies to establish whether the children tolerated the medication and whether improvement was sufficient to warrant more stringent, blinded studies. The open label studies were followed by double-blinded comparison studies. We completed the study cycle with an open label portion to give the children who did receive the medication an opportunity to receive it and also to have a nine to twelve month exposure to learn about continued efficacy and tolerance.

Segment I studies were typically 30 to 90 days in duration with first doses about 50% of maximum dose. Dosage was increased to tolerance over a 2 to 4 week interval depending on the length of study. SAARD studies usually called for a single dosage for 6 months with increased dosage afterward if necessary. The medications were almost always from the pharmaceutical companies. Some studies were funded by the NIH and were prepared separately. Detailed and standardized forms were used and monitoring was performed. Dosages were in meter-squared calculations in the early studies and mg/kg in some studies.

Joint examination techniques were carefully studied and the clinic directors all tested each other to be sure we called joint changes in similar ways. However, fundamental to success was the recording of the change in joint function at later visits to establish improvement or worsening of joint manifestations of swelling, tenderness, loss of flexion or extension. The change in joint status was the critical measurement. The same examiner judged each visit. Thus the error factor of subjective judgment was nullified because the same error would be constant. Another strong feature was that experienced clinic directors made all of the evaluations.

Segment II studies were designed to study efficacy and safety. The model was to compare the study drug to aspirin in a double-blinded manner. This meant a mechanism was necessary to blind not only the patient and family but also the clinic director caring for the patient. We did this by sending coded meds to the director from our coordinating center or the pharmaceutical company. Later, in a SAARD study, a pharmacist at the participating center filled the prescription with a coded prescription. Both methods worked successfully for us. Monitoring was done mainly by the pharmaceutical company monitors who made frequent visits to the site. Detailed data was collected and analyzed. The data analysis was the product of Ed Giannini's genius. The interested reader can retrieve many publications of our data analysis techniques. Ed also was the key person in supervising data collection and compliance.

From the beginning we insisted on collecting our own data and analyzing it for publication. This concept led to some memorable battles with individual drug companies. They wanted to interpret our data for the FDA. We compromised by using our data for our publications, and they analyzed the same data for their own purposes. The data for FDA approval was our data as it turned out in later studies. Our credibility and data became the gold standard for studies in children with arthritis.

## Explosive growth of the PRCSG

The FDA section devoted to anti-rheumatic drugs under the supervision of Dr. John Harter became a friend of children with arthritis. John suggested to drug companies that they should now study anti-rheumatic drugs in children because it was fair and the right thing to do. He insisted that approval of the drugs for adults where the financial incentive was paramount required studies in children also. The effect was immediate. McNeill had also received favorable mention for their pioneering venture with us. We suddenly had a host of companies asking for our services to study drugs in children. Our good fortune was that we had labored hard to develop a methodology to study these new drugs that were being called NSAIDs. Initially they were thought to be for arthritis alone, but it was apparent to all of us involved that the application was universal for pain relief and anti-inflammatory effect.

We soon had contacts and offers from Eli Lilly, Parke-Davis, Sandoz, Ciba-Geigy, SmithKline, and several other companies. The effectiveness of NSAIDs for pain relief and reduction of inflammation gave great impetus to drug companies to bring to market these compounds quickly. In the early days when the push was for arthritic use alone, I became aware of the generic pain relief when nursing staff asked for the samples of NSAIDs for personal relief of menstrual cramps.

We quickly became the only game in town for children's studies, and we labored to fulfill the demand for studies. This enabled us to have a wider selection of medicines for children with arthritis. We performed studies on tolmetin, naproxen, fenoprofen, meclofanamate, pirprofen, proquazone, ketoprofen, oxaprozin, ibuprofen, and others. Some drugs did not pass muster after a Segment I study because of either unpleasant side effects, poor efficacy or the drug company felt they had done their duty to the FDA. Other than aspirin and one or two other exceptions, most of the NSAIDs studied had virtually the same efficacy and toxicity.

Many new organizational concepts were necessary for our group. We were literally the blind leading the blind. We had no template to follow. We decided not to incorporate. This meant that each center had to make a separate subcontract with a sponsoring drug company for local payment. We were the coordinating center in Houston and the contracts were negotiated by us. It was impossible to have a set amount for each test and each clinic charge. The variations in laboratory charges in the USA were mind-boggling. Price differences more than 800% in certain tests were unbelievable to me but true. The solution was to have charges paid to each institution rather than try for uniformity of amounts paid. We did pay the same amount to each investigator for a visit and the same amount for the research assistant support. In a few later studies the drug company contracted with a central laboratory with all specimens sent to that lab by mail. We had problems obtaining results in a timely manner from the lab. As a cost containment device, it was not satisfactory. The advantage was that we had comparable data with less error factors because the same lab was doing the tests.

Jerry Jacobs at Columbia in New York City always had the highest fees (everything in NYC was higher). Jerry was our good cop/bad cop and harassed the monitors of the drug companies with incredible complaints. Several companies refused to deal with him and required me to be the intermediary; however, Jerry may have complained the most, but he always finished what he promised and always did it well. Most of the time his complaints were absolutely correct and helped make the PRCSG stronger and more respected.

The coordinating center in Houston was crucial to the projects. The other members were concerned that I would be paid more money. There was no understanding of how much work was required to conceive, organize, and supervise the studies. After I retired, the money to support coordination was finally corrected. With the addition of Ed Giannini's skills in study design, data collection and statistics we added considerable efficiency to our efforts, which were quickly recognized nationally. Inferential and descriptive analyses were applied to the data that were acceptable and understandable to both clinicians and statisticians. In the 1970s Jane Schaller and I became a part of the Advisory Committee of the FDA Anti-inflammatory Group. Children had a seat at the table. We participated in the formulation of the study guidelines for NSAIDs.

The study of SAARDs (slower-acting anti-rheumatic drugs) by the PRCSG came in the early 1980s. They required modification of our basic Segments I, II, and III studies but were essentially the same as the NSAID studies. This addition to our program came as a result of the interest by the National Institutes of Health in the PRCSG and our efforts to study drugs in children. This need coincided with the NIH program of cooperation with the USSR in medical studies during the cold war.

Two early hopes For PRCSG studies: In addition to the main purpose to provide approved drugs for children with arthritis, my hope was that our use of identical study methods would allow comparisons of drugs in a realistic manner. We were able to do this with a number of studies. I also hoped that we could build a database of studied aspirin patients and use them as historical controls. We could thus study a drug without a blinded control. Alas, the first two Segment II studies comparing tolmetin to aspirin and fenoprofen to aspirin were not similar enough to justify elimination of blinded studies.

Four observations:

1. Data entry and analyses were performed better by our own data processing unit than by each drug company's IT personnel. The firms were too busy with other studies to process data and report in a timely manner.

2. Most of the firms entering the area of NSAID and SAARD studies were usually inexperienced in this area. Even more importantly, we had to reinvent the wheel with each drug company. Each new company supervisor had to present his/her ideas of how the forms should be done and how the data should be treated. It was always a painstaking exercise to persuade them that we had our own methods and data analysis. They always came to the same view that the standardized, proven methods of our group were better than reinventing the wheel, but it was always difficult and time-consuming.

3. Even though FDA personnel were sympathetic to the need of children for these medicines, FDA approval to use these drugs in children proved to be an elusive and time-consuming exercise. There were people who pushed for safety and dosage testing only with no provisions for efficacy testing. For a period of years there were shifting goal posts for approval.

4. The expense to bring a drug to market was and is outrageous. About 40 million dollars were needed in the 1970s and 1980s. Now in the 2000s, several hundred million dollars are necessary. The patent law rules of a 17-year window from patent to end of patent protection were unreasonable in the old days and are still unreasonable. It usually required seven to ten years or more to gain FDA approval for a given drug. This resulted in a drug company needing to charge enough to recover outlay expense in only seven years. One can argue the problem in many ways, but the result was and is outrageous prices for drugs. The drug companies in recent years have not covered themselves with honor and glory in pricing drugs differently in the rest of the world than here at home.

## Reflections on the first nine years of the PRCSG

I published an editorial on our drug trials in the Journal of Rheumatology in 1982, much of which has just been discussed [2]. Additionally, our small collegial group was working on the JRA criteria, striving for a place at the table with adult rheumatologists, trying to be heard by the American Academy of Pediatrics, and attempting to persuade the medical schools to include rheumatology in the pediatric department curricula. Our task was daunting. The FDA was a pleasant surprise. Dr. John Harter believed, as we did, that children with arthritis deserved to have drugs to help them. Our success was due in large part due to this recognition. Our methodology was cutting-edge and became a gold standard for anti-rheumatic drug studies in children.

A major concept that we fought to make an integral part of studies was the double-blinded study of drugs. A few of our small group felt that double-blinded, placebo studies with allowance for a base NSAID were unethical. Jane Schaller was the most concerned. When we planned our first blinded SAARD study using gold, d-penicillamine, and placebo, Jane objected. These concerns spread to Norway when one of the highest officials of government held special hearings on the ethics of the concept. We prevailed thanks to an enormous effort by Dr. Hans Martin Hoyeraal of Oslo. Interestingly, it turned out that the children who received the placebo were the safest because neither penicillamine nor hydroxychloriquine were really effective. The children not on the drugs had no exposure to toxicity. At these times the placebo was the safest drug to use.

The FDA connection was in some ways a double-edged sword. After a few years, it was apparent to several of us that a large number of "me-too" drugs were coming to market. The NSAIDs were remarkably similar in action and toxicity. There were a few differences such as the significant liver toxicity of aspirin as well as its ability to produce gastrointestinal bleeding. Most of the other NSAIDs were markedly less with regard to these toxicities. Indomethocin was a sleeper drug for toxicity and did cause serious GI bleeding. The basic question was how many NSAIDs should be studied. I initially thought that we should not study so many of them. Two events changed my mind. All children did not respond to a given NSAID. Karyl Barron, Don Person, and I reviewed over a hundred patients who received more than one NSAID and 79 had toxicity. About one-half of these children had toxicity to the second drug, and almost two-thirds had a similar toxicity. We therefore needed more NSAID options for children with arthritis. The second event was finding that efficacy for one child is not necessarily the same for another child. We had children who had marked improvement on a certain NSAID and no improvement on another. These selected differences were not apparent in studies of children in a single study but were noted only after a large number of the NSAID drug studies were completed.

The late 1970s were years of epiphany for our group. Several important facets of our progress came into being or to fruition at this time. The FDA included me in their Arthritis Advisory Committee where I was chair of a subcommittee to write guidelines for the study NSAIDs. The NIH included us in the USA-USSR scientific cooperative studies. The future and fate of the PRCSG became totally entwined with the NIH and the FDA from these years onward. The NIH sponsored the adult group under Dr. John Ward's Chairmanship to do cooperative studies in adults with arthritis. John was Professor of Medicine at the University of Utah and my friend. In fact, John, the FDA, and the NIH wanted me to move to Salt Lake City where the adult and pediatric study programs could be joined and supported by the NIH. It almost came to fruition. My wife Ria and I built a house in Park City, Utah in 1975 and in 1977 I successfully took the Utah medical board by oral exam. I was on the advisory committee of John's Cooperative Studies Group. Ria's parents became seriously ill in the late 1970s, and we decided to stay in Houston.

## V NIH-USA-USSR scientific cooperation program 1975

An apparently unrelated pivotal event for pediatric rheumatology occurred on May 23, 1972. President Richard Nixon and Secretary Leonid Breznev of the USSR signed an agreement of scientific cooperation. The purpose was, of course, to cooperate in areas that posed no threat to either nation during the cold war. The thesis as presented to me by Dr. John Decker of the NIH was that we would either learn to work together or we would die together. Children with arthritis turned out to be the major point group for this concept. This event changed the course of pediatric rheumatology and intertwined the PRCSG, pediatric rheumatology, the FDA, and the NIH into a cooperating functioning partnership that propelled our cause into new levels of acceptance and effectiveness.

As I understand the story from several participants, Secretary Weinberger of HHS was asked by President Nixon what disease he would choose as the first thrust of the scientific cooperation. Weinberger's wife had severe arthritis, and he suggested it. Fortuitously, the USSR's strongest medical specialty was rheumatology and had been since the days of Stalin. Professor Nesterov was Stalin's physician. He was a rheumatologist also. Thus, compared to other fields, rheumatology became a favored specialty for funding over many years. Spin forward quite a number of years to the1970s. Professor Valentina Nassonova was Secretary of the Academy of Medical Sciences (AMS) of the USSR (in the Soviet system the secretary was the leader of a group or committee) as well as Chair of the Institute of Rheumatology of the USSR. The AMS owned all of the research institutes of the USSR and reported directly to the Supreme Soviet (their Congress) rather than to the Ministry of Health.

The Russians wanted to study arthritis also -no surprise. The first meeting was at the Stone House of the NIH in May 1975. There were three Russians: Nassonova, Dr. Margarita Ivanova, and Professor A. Speransky.

**Figure 2:**
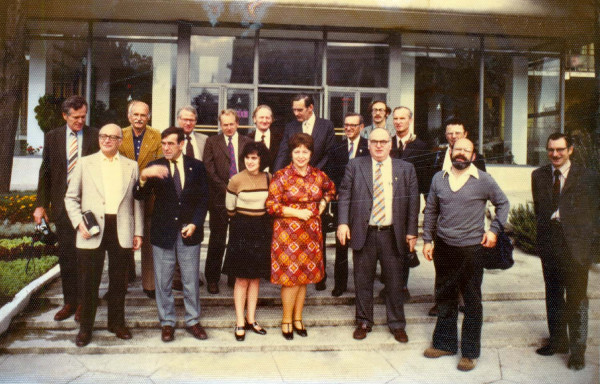
**NIH USSR Visit 1976**. From left: Drs. Charles Christian, Manuel Rudd, Ralph Williams, Lawrence Shulman, Israel Jaffe, 2 Russian hosts, Alphonse Matulis, Russian host, John Decker, Morris Ziff, Fred Steinberg, Gerald Rodman, John Mill, Robert Swezey, John Baum, Lev Alexeev. Earl Brewer took picture.

**Figure 3:**
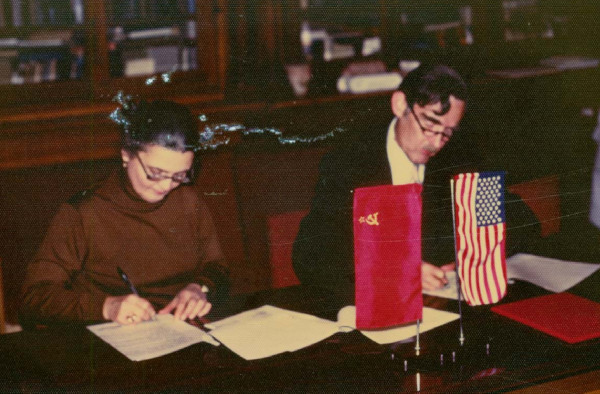
**USA-USSR Signing agreements 1976 -Moscow**. From left: Professor Valentina Nassonova, USSR Academy OF Medical Science, and Dr. John Decker, USA-NIH signing Memorandum of Understanding 1976.

The initial contacts went well in 1975 and 1976. [Figure 2] [Figure 3] The members of both groups felt at ease with each other and were honest with each other at all times. Basically our Soviet counterparts were the same as our group. They were interested in the welfare of children and families and wanted to provide the best care for them. In this way medicine has always been so universal with great commonality of interest and personality the world over. As individuals they were also interested in achieving as much success as possible in the terms of their system. Most were not very politically minded. In achieving our mutually agreed goals for our studies, each of us carefully discussed how we could work each of our systems to get permission to do the studies to help the children and their families.

**Figure 4:**
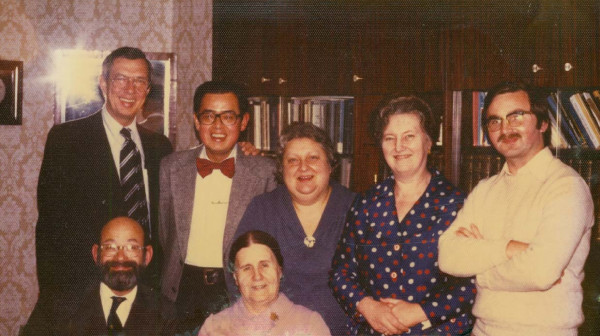
**1976 USA-USSR Pediatric rheumatology meeting – Moscow**. Front row from left: Dr. John Baum and Professor Alexandra Dolgopolova. Back row from left: Dr. Earl Brewer, Dr. Andrew Kang, Professor Alexandra Isaeva, Professor Nina Kuzmina, and Dr. Alexandr Shaikov.

The late Professor Dolgopolova was the original director of Pediatric Rheumatology in the USSR. [Figure 4] She was truly one of the great physicians of our time, and I still miss her integrity, intelligence, humility, sense of humor, and can-do, problem-solving abilities. She and Professor Nassonova were two of the best problem-solvers I have ever encountered. In my initial discussions with Professor Dolgopolova in 1976, she stated that the results of our initial study of the epidemiology of JRA in the USSR-USA, including a five-year follow-up of patients, must be published in scientific journals of both countries. I explained to her that in the United States, we would prepare the paper to both our satisfactions and submit it to the editor of an American journal who would review the paper for approval as to quality. She then replied. "Da, it is the same here in the USSR – Of course, I am also the editor." She then asked about medical specialty training in the USA. I explained that two to four years of training were customary and that the candidate had to pass an examination given by a board to establish competence. She smiled and said, "Da, it also the same here – Of course, I give the board examination."

Our first study did not involve medicines for arthritis. Rather it was a joint effort to establish that JRA was the same entity in both countries and to ascertain the outcome after five years or more in both countries. John Baum of Rochester, Professor Dolgopolova, Lev Alexeev, and I were responsible for the study. One center was used in the USSR and five centers in the USA. Publication was in Arthritis and Rheumatism in September 1980 [3] with a companion publication in the comparable Russian journal. We established that JRA is the same in both countries and that the long-term course of disease is about the same in both countries.

The second study of two anti-rheumatic drugs, d-penicillamine and hydroxychloroquine, tested against placebo resulted in uncharted territory involving the Pharmacological Committee of the Ministry of Health, USSR. We also had the need for substantial funding from the NIH as well as permission to do the study from the FDA. There was considerable discussion on our side about whether we should give the Soviets our expertise in good drug studies. There was also a disdain by certain factions at the NIH who argued that the time and money were a waste of the expertise of the NIH and should be paid for by the State Department and not the highly sophisticated NIH. With a great deal of pressure from John Decker and several of his colleagues at the NIH, a contract was awarded to the Pediatric Rheumatology Collaborative Study Group with me as principal investigator. The contract was awarded only for the USA side, however, and certain powerful people in the NIH instructed me directly and indirectly to not even mention the USSR side of the study. At the end of the study, I simply ignored the orders and wrote a report with Dr. Giannini and group, published in the New England Journal of Medicine (NEJM) in May 1986 [4]. It was presented as the number one paper at the American Rheumatism Association (ARA) Meeting in Los Angeles in 1985. The study took 8 years from first planning to publication. A total of 162 USA-USSR children participated in the one-year study. This marked the first time that the Soviets requested permission from the NEJM to publish essentially the same paper in a Russian journal Rheumatology.

Even more intrigue occurred in obtaining permission to do a study of auranofin (oral gold) and placebo in children with JRA (231 USA-USSR children) combining the efforts of a pharmaceutical company, the NIH, and the Soviets. The last event was to do a study of methotrexate and placebo in children with JRA combining the PRCSG, the FDA, the NIH, a pharmaceutical company, and our colleagues in the USSR. The FDA had not worked with the USSR before, and with reason, many people were worried. To everyone's credit the problem was solved with only two PH (pigeon hole) syndrome expeditions at the FDA necessary.

Our Soviet counterparts had even more trouble. The head of the Pharmacological Committee of the USSR (the Russian equivalent of the FDA) went head to head with Professor Nassonova of the Academy of Medical Sciences over turf. He maintained with justification that any blinded, research drugs should come under his supervision. Nassonova maintained that the Academy of Medical Sciences of the USSR had made agreements with the National Institutes of Health of the USA directly, and the subject matter was none of his concern. The control problem continued through our last three studies. The crux of the argument was that the USSR Ministry of Health and Pharmacological Committee wanted to go into the drug study business. They could bid about one-third of the usual cost in the USA for a comparable study. The problem was that no one was buying. They needed credibility. Hence, the turf war resulted. Nassonova and I pointed out to them that under the terms of the bilateral agreements each must bear the cost of a study in its own country. Therefore it was impossible for the USA to pay for the expenses of the studies in the USSR.

We dealt with three different officials of the Ministry of Health for each study. Professor Lepachin of the Pharmacological Committee stymied the first drug study of d-penicillamine and hydroxychloroquine. Lepachin had impounded the study drugs at the airport. It was solved on this occasion when Nassonova and I liberated the study drug at the airport in Moscow. It was a great lesson in gutsy diplomacy watching Nassonova stare down the Customs Director at Sheremeteva Airport.

The second study of drugs was auranofin and placebo. This time, Professor Babayan, an Armenian at the Ministry of Health, impounded the drug by taking them to his office at the Ministry to be sure that we did not liberate them again. Nassonova, Kuzmina, Shaikov, Alexeev, and I met with him on a cold December day in Moscow in 1984. After much discussion he agreed to let the study progress with the understanding that the data would not be released until the SmithKline Pharmaceutical paid a registration fee. We did the study and it was reported in both the USA and USSR in 1988. We still have not been told how to register the drug.

Professor Pokryshkin replaced Babayan, and in July 1987, Nassonova, Shaikov, Kuzmina, and I went head to head with Pokryshkin. He placed us around the conference table with me on one side and all the Russians on the other side. They spoke Russian for about one half hour with a lot of nodding and pointing. Nassonova early on pointed to Shaikov and said, "Alexandr, Professor Brewer is over there by himself. Sit with him and translate." Finally Pokryshkin suddenly smiled and said in perfect English. "Ah, Professor Brewer. I finally understand the problem." Nassonova smiled and said, "Professor Pokryshkin had forgotten that it is unethical for us in the USSR to accept money for the study of drugs to help our children. It is quite impossible."

Thus the study of methotrexate was tentatively approved. However, Professor Lepachin was not through with us yet. Professor Pokryshkin was reassigned with a new reorganization at the Ministry of Health. Professor Lepachin, who had been my friendly adversary for those many years, remained the Chairman of the Pharmacological Committee. We have never met to this day. His next and last move was to convene a meeting of his committee and ask several very pertinent questions about the drug. Dr. John Harter and Dr. Kent Johnson of the FDA, Dr. Jack Klippel of the NIH, Dr. Margaret Gandt of Lederle Laboratories, Dr. Giannini, and myself addressed these issues with an appropriate letter sent by Dr. Marlene Haffner, Director of the FDA's Orphan Products Division. Finally on January 13, 1989, the study of methotrexate in the USSR was approved and the medication was shipped without event and the study and was completed October 1, 1990 and published in the New England Journal of Medicine in 1992. In an article published in the British periodical, Nature, March 19, 1987, Dr. James Wyngaarden, Director of the National Institutes of Health, stated that the methods developed by the USA-USSR pediatric rheumatology scientific cooperative group had pointed the way for the expanding NIH research efforts with the USSR.

## The Giannini years with the PRCSG – 1976

I don't remember the first time that we met, but Ed was completing his doctorate of public health at the University of Texas School of Public Health. He came to work for me part-time to help with our data. Ed was a larger-than-life, effervescent, assertive, happy Italian-American who burst into a room and took over. He brought enthusiasm, efficiency, and competence to our efforts. At that time he was single and also our Casanova-in-residence. We lived vicariously through Ed's adventures. His knowledge of computers and data filled a void and allowed the PRCSG to blossom, and blossom it did. Over the next decade or so we studied 15 or more drugs in various segmental studies.

The PRCSG had reached a turning point when Ed joined. We moved to the next level of organization of our collegial group of eight members (Brewer, Baum, Cassidy, Fink, Hanson, Jacobs, Levinson, and Schaller). We embedded Segments I, II, and III methodologies into a codified, written system. All studies started and ended with the standardized methodology. We also wrote and approved by-laws and rules and regulations whereby the chair was elected along with a few center directors. Later we added members from the FDA and the NIH. The system worked well with a few bumps along the way. We established our credibility with our peers, the then-ARA, the FDA, the NIH, the drug industry, and the AAP.

**Figure 5:**
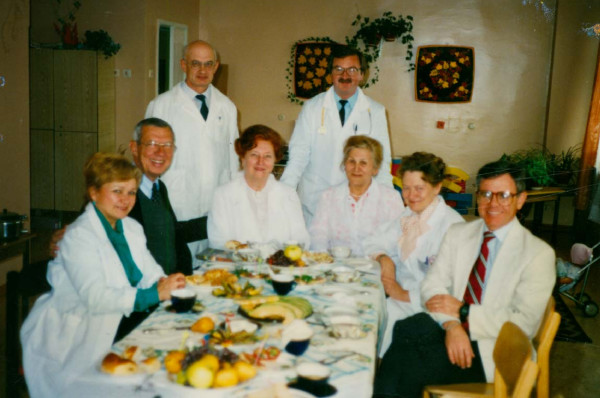
**1978 – Moscow – Russian colleagues and Giannini – Insitiute of rheumatology**. Standing from left: Drs. Boris Shokh & Alexandr Shaikov. Sitting from left: Dr. Margarite Ivanova, Dr. Earl Brewer, Professor Nina Kuzmina, Dr. A. Yakovleva, Dr. Nina Melikova, and Dr. Edward Giannini.

Ed quickly became essential as our senior scientist. He was our coordinating operating officer for data and customized software to fit our needs for data processing and form development. [Figure 5] The PRCSG held frequent meetings, usually before and after different studies. We hammered firm and standardized methods of examining patients, how to pay each center, and data processing. We held firm to our agreed methods when several agencies or people tried to change our mission. We published our concepts and methodology in a number of journals and books.

In the middle or late 70s, I came to the mistaken conclusion from organizational data supplied by allegedly learned people that no more than six clinics could do joint studies together efficiently. We were in the process of approving several of the fifteen studies that we performed, and I thought that adding a Group II would double our capacity for studies. We asked Jack Bass of Ohio State Medical School in Columbus to be chair. Other members were Balu Athreya at Children's Hospital of Philadelphia, Donald Goldsmith at Temple in Philadelphia, J. Roger Hollister at the Jewish Hospital in Denver, Deborah Kredich at Duke in Durham, North Carolina, John J. Miller at Stanford Medical School in Palo Alto, and Lauren Pachman and Ken Rich at Northwestern Medical School in Chicago. Previously we decided to add colleagues at several of the original centers to help with the studies. They were Harry Gewanter in Rochester, NY, Bram Bernstein in Los Angeles, Donita Sullivan in Ann Arbor, Michigan, Norma Battles in Dallas, and William Crowe in Cincinnati. The arrangement was short-lived. Group II did one or two studies before it became apparent that the plan did not work. Instead of promoting efficiency, we added a layer of bureaucracy that hindered our mission instead of helping. Jack Bass visualized that the new unit would be a separate entity, which would function independently of the PRCSG board. We abandoned this concept in a few years.

Our participation in CLINFO began in the late 70s or early 80s. This was the precursor of the Internet developed by the Defense Department first and then the NIH to connect research centers together by computer to facilitate interchange of data and ideas. Ed became a part of this project while working at Methodist Hospital during his student days. He included the PRCSG in the program. They even ran an underground cable to our offices to connect us. In a few years Ed was selected to help write the programs to adapt CLINFO mainframes to the personal computer. We were in the mainstream of progress in data research and processing.

Ed and I fought many battles with a variety of interested groups concerning study methodology. One faction felt that children were little adults, and dosages could be extrapolated with no concern for the problems of growth and changes in a developing body. The cost of adding children to adult studies was the driving force. The market for children was small for the rheumatic drugs, but it became clear to many of us that NSAIDs were going to be needed for pain and anti-inflammatory effect in many childhood conditions.

I have discussed the problem of placebo studies earlier. Ed developed data treatments that were on the cutting edge for our group of studies. He was clearly the glue that bound the core of the PRCSG. When I retired in 1990, I asked the group to continue Ed as Senior Scientist and Dan Lovell as the Chair of the PRCSG with headquarters at the University of Cincinnati. The board and membership agreed and elected both. The PRCSG has moved to new levels of sophistication far beyond our original beginnings with more than seventy centers now in the USA, a European cooperating component, PRINTO, and fifteen pediatric rheumatology centers in China led by Dr. He Xiaohu in Beijing that may become a part of the group.

## VI Pediatric Rheumatology Council on Pediatric Rheumatology – ARA-1976; Park City pediatric rheumatology meeting – 1976 – Park City I

Laurence Schulman and Gerald Rodnan, both presidents of the ARA, appointed the first Pediatric Rheumatology Council in 1976. The initial members were John Baum, James Cassidy, Chester Fink, Virgil Hanson, Jerry Jacobs, Joseph Levinson, Jane Schaller, Sydney Stillman, and myself. The idea for the first PC meeting came from this group, and the members became the program committee for the meeting. The council was the natural outgrowth of the JRA Subcommittee.

The Park City meeting from March 21-25, 1976 was another pivotal breakthrough for our subspecialty in the US and worldwide. Parallel events were moving so rapidly that one has to place this seminal meeting in perspective with the other rapidly moving events. The PRCSG was hurtling forward in its destiny. We were just beginning our studies with the Russians. The ARA recognized pediatrics by forming a council on pediatric rheumatology. The JRA criteria validation was on the road to being published in 1977.

I think that the original thrust probably came from the ARA council meeting. Virgil Hanson and Jane Schaller were co-chairs. Ria and I were building a house in the then-undiscovered Park City ski resort town in Utah. We met at Marsac Lodge at the Park City Ski Resort the first time and later meetings were at our new house. In addition to Jane Schaller and Virgil Hanson, initial members of the committee were John Baum, James Cassidy, Chester Fink, Jerry Jacobs, Joseph Levinson, Sydney Stillman, and myself. We obtained seed money from the Shriner's of North America and the Lazarus Foundation. This was essential early seed money. Larry Shulman of the NIH also helped.

We had a number of committee meetings. Jane was selected to be editor of the papers presented. Barbara Ansell and Eric Bywaters of London were prominent participants in absentia. Egos and alter egos were prominent. Our collegial spirit prevailed. Sydney Stillman was a strong guiding factor. Syd had no interest in skiing or anything resembling it. We met in the Steeps room at the Park City resort. Each morning Syd was early to rise for a brisk walk in his tweed coat, white shirt, tie, slacks, and formal Boston hat. His fellow walkers were headed to the gondola in ski attire. He was wonderful. He had a cheery hello for everyone he met.

Arthritis and Rheumatism agreed to publish the papers of the meeting [5]. There had been nothing like it before. We selected the Treasure Mountain Inn, a charming, old mining inn on historic Main Street for +the meeting place. The meeting room had a large fireplace with 1890s furniture (Park City was a 1890s mining town), and easily held the fifty-eight or so participants invited to the meeting. We ate our meals at the Car 19 restaurant across the street. We held our final banquet in the basement rock room of the Car 19.

Everyone felt the excitement of the meeting. We knew that this was a major breakthrough. We agreed to have several series of patients with data at the beginning. The program was truly a compendium of knowledge relating to pediatric rheumatology. Three series were presented. Clinical aspects of JRA including heart, joints, epidemiology, immunology, and radiographic aspects were included. The other rheumatic diseases were also covered. Treatment and family and patient education received prominent roles. It was amazing to me to look at the breadth of information. The Arthritis and Rheumatism publication of the meeting in a Supplement in 1977 served as a reference of pediatric rheumatology for the next generation [5].

**Figure 6:**
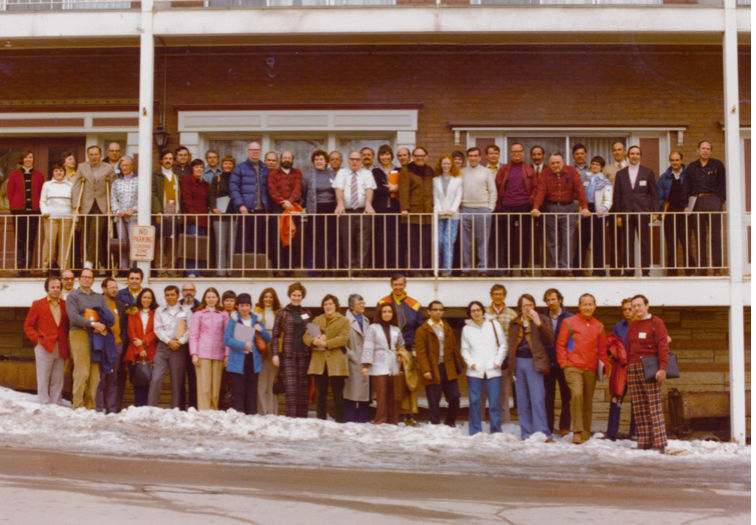
**Park City I meeting 1976**. Fifty-nine participants pictured at Treasure Mountain Inn, Park City Utah, 1976.

A major highlight of the meeting for me was the wide-angle color picture of all fifty-nine of us on the balcony of the Treasure Mountain Inn taken at the end of the meeting. [Figure 6] Jim and I resurrected it for subsequent meetings. It is a classic picture of some of the pioneers in our field.

Professor Eric Bywaters from London and Taplow was certainly our most distinguished and honored guest. He had a remarkable ability to make everyone he met feel at ease. The sly smile, his horn-rimmed glasses, and a slight ducking of his head when making a point made his charisma even more potent. He was slight of build with thinning hair. Eric gave our main, after-banquet talk at the Car 19 restaurant on Main Street in Park City. His discussion of the early observers of children with arthritis was clear and wonderful. Eric, ever the gentle consensus builder, gave his take on the initial pioneers who made pediatric rheumatology an entity. His comments are classic and are recorded here:

"I think I saw it arrive, although I cannot specify its birthday or place and I am damned if I can read the father's signature on the birth certificate. We are very fortunately a small enough group to know each other personally and to cooperate and enjoy each other's company."

### PARK CITY II – 1986

Many events moved in concert acted to create a golden era for our cause. Park City II occurred ten years after the historic Park City I, and a comparison showed how far pediatric rheumatology had progressed. The meeting was held March 15-19, 1986 in Park City, Utah and sponsored by the Pediatric sections of the AAP and ARA, the AJAO of the AF. The committee members were Balu Athryea, Earl Brewer, James Cassidy, Virgil Hanson, and Bernard Singsen. Three of the five members were members of the first PC-I committee (Brewer, Cassidy, and Hanson). James Cassidy and I were co-chairs. There were 17 invited papers and 56 peer reviewed abstracts. Three distinguished, retiring or retired pediatric rheumatologists were honored at the meeting – J. Sydney Stillman, Joseph Levinson, and Virgil Hanson. Each gave memorable talks at the meeting. Syd gave his thoughts on the beginnings of pediatric rheumatology to the 1930s, when small groups at the Royal College of Physicians tried to stimulate research in rheumatic diseases. He left this history to others and focused on our American efforts.

"Thirteen physicians including two Nobel Laureates in 1932 held the first meeting of the American Committee for the Control of Rheumatism. In five years, it became the American Rheumatism Association (ARA). I first attended the seventh meeting of the ARA in June 1940. Only five papers were presented. Three (60%) of the papers were devoted to children with rheumatic disease. One of the papers was by Dr. William Green of the Children's Hospital in Boston, His subject, "Monoarticular and Pauciarticular Arthritis in Children."

Rheumatic fever was a major cause of rheumatic disease in the first half of the twentieth century. The House of Good Samaritan at the CHMC at Harvard treated rheumatic fever for over 40 years from the 1920s to 1960s. In 1921 the first 100 cases, admitted with their first attack, had a five-year mortality of 24 percent. By the 1950s the mortality was only 3 percent. Many reasons were given for the decline: decrease in virulence and epidemicity of the infecting strains of Group A streptococci, improvement of living conditions, and most importantly, the advent of penicillin. By 1960 the House of Good Sam as it was called was closed for lack of patients. Syd reviewed the important events propelling pediatric rheumatology forward. His parting words and advice were profound and useful to everyone. "I should like to think of the field of rheumatology as it was when I entered it. It was an association formed for the mutual aid and protection of its members, and for the furtherance of some common purpose. The guild was small enough that one knew most of the members, both here and abroad. There was cooperation in planning meetings, arranging for training fellows, and sharing information. When one of your patients was traveling, you knew you could refer him or her to a good physician almost anywhere. Now that I have retired, I find the warm relationships built up during my active years still persist. I can give you no better advice than to be a good, participating, generous member of the guild as we work together toward a solution of the difficult problems presented by the rheumatic diseases."

Joe Levinson's reflections and perspectives were equally profound to me. He said that looking back over thirty years, we had few tools in our armentarium – only a few drugs, few helpful surgical interventions, unproven and controversial physical programs, but a lot of faith. We also had a great deal of clinical curiosity, so we systemically studied the children we treated. We sought to describe and classify disease. We tried to visualize the relationships of spectrum of disease and comprehend relation to varied responses to common exposures. We searched for understanding of both technical and emotional roles we played in the lives of children. Classification was only a starting point for predicting functional outcomes for individuals because coping strategies, expectations of the families, and perceptions of the disease and child by schools and other community agencies were just as important. We had to develop teams as part of the patient's extended family to develop realistic expectation of performance.

The progress in 10 years was breath taking to those of us who were there from the beginning. Fifty-eight professionals participated in Park City I in 1976. Two hundred professionals attended Park City II. In 1976 there were 17 pediatric rheumatology clinics and 22 pediatric rheumatologists in the USA and 4 clinics and 13 pediatric rheumatologists Canada. Ten years later the number of clinics increased to 71 and pediatric rheumatologists to 103. In Canada the numbers increased to eight clinics and 13 pediatric rheumatologists. Nine peer reviewed pediatric rheumatology papers were accepted for presentation at the ARA meeting in 1975. In 1985 the number accepted increased to 35.

### Other Major Conferences: The EULAR Conference in 1977 in Oslo

**Figure 7:**
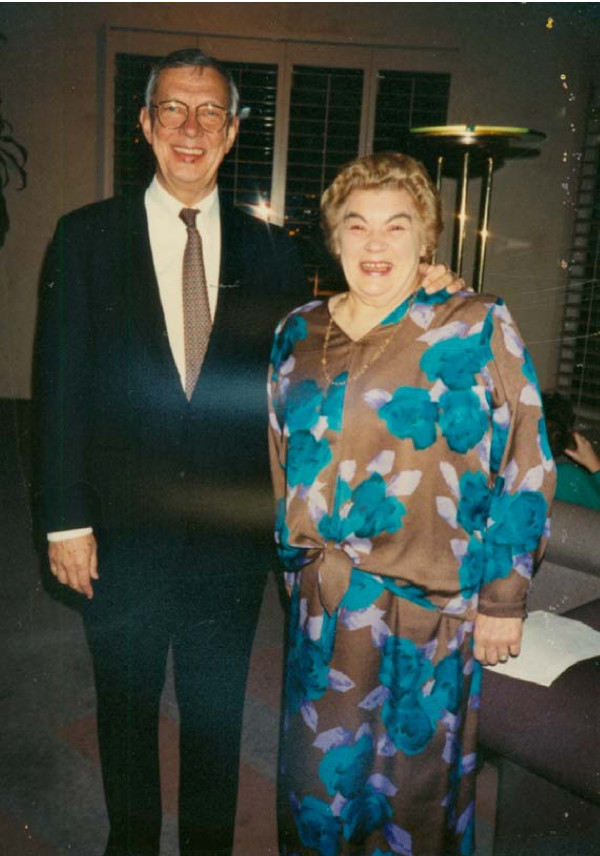
**Park City III 1991**. Dr. Earl Brewer [L] and DR. Barbara Ansell [R].

This conference was a pivotal event that firmly established our USA connection with our colleagues in Europe and revealed the deeply felt differences of approach, treatment, and classification. Eimar Munthe of Oslo was our host, and the symbol of the meeting was the crying child sculpture by Viegland. It was here that Barbara Ansell, a loved and revered legend of pediatric rheumatology, [Figure 7] and colleagues passionately pushed their feelings about classification. Essentially they wanted to rename JRA, Juvenile Chronic Arthritis and wanted to do it by executive decision and not by detailed studies. We felt that too much effort had been expended in validating our American ARA studies to change. Also Ansell and Phillip Wood wanted to add ankylosing spondylitis, psoriatic arthritis, and later enthesitis.

There were two major forces at work. Barbara wanted to pursue her goals for development of pediatric rheumatology through EULAR in Europe. She did not want the PRCSG to form a third unit in Europe. I tried to add Hoyeraal in Oslo, Anna Lisa Makeli in Finland, and others in Europe to our American PRCSG group. The correspondence in the papers in my archives is fascinating. Phillip Wood and Barbara Ansell suggested that the pediatric rheumatologists in Europe who had signed up for the PRCSG withdraw and asked the PRCSG to work exclusively through the EULAR organization. In my opinion, this move may have delayed cooperative drug studies in Europe until the Pediatric Rheumatology International Trial Organization's (PRINTO) birth years later.

### The Eightieth Ross Conference on Pediatric Research in 1979

This meeting was a project led by Chester Fink. Chet had several laudable agendas that he wished to promote. The first agenda was to persuade Ross Laboratories to sponsor the next Park City pediatric rheumatology meeting. The Ross Conferences were highly prestigious in the pediatric world at that time. This sponsorship would give us access to over 30,000 pediatricians for educational purposes to promote better understanding of children with arthritis. His next agenda was to get the Park City meetings away from the snow. He and other members of our small collegial group had no interest in skiing or cold weather. We therefore held the Ross meeting in the Bahamas at the Xanadu Hotel, formerly the headquarters of Howard Hughes. The quality of the meeting was a great success and the publication of the meeting served as another pediatric rheumatology reference. Unfortunately, the accommodations were not the best. While Cathy Hanson was drawing a bath, the hot water faucet stuck in the on position. Water ran over the side of the tub, into the room, and into the hall. She was forced to get help with few garments on her body. The stories went on and on. We returned to Park City in1986 for Park City II.

## Conclusion

The 1970's and early 1980's were an exciting time for pediatric rheumatology both in the US and throughout the world. The growth of our subspecialty from a few to many was spectacular and our collaborative efforts in classification, meetings, drug studies both in the US and abroad, and organization of our field were no less impressive. I was delighted to have some role in these endeavors and am happy to be able to describe these historical, pioneering steps in this three-part article. Soon-to-be-published Part III will relate our early efforts to receive support for pediatric rheumatology from the US government, develop a section in the American Academy of Pediatrics, begin a board certification process, and train fellows to carry on our important work.

## Abbreviations

AAP-American Academy of Pediatrics

AMS-Academy of Medical Sciences of the USSR

ARA-American Rheumatism Association

CLINFO-US Defense Department Computer Project to connect medical centers in the US in the 1970's

FDA-Federal Drug Administration in the US

NEJM-New England Journal of Medicine

NIH-National Institute of Health in US

NSAIDs-Non-steroidal anti-inflammatory drugs

PH-Pigeon hole syndrome

PRCSG-Pediatric Rheumatology Collaborative Study Group

SAARDs-Slower-acting anti-rheumatic drugs

## Competing interests

The author(s) declare that they have no competing interests.
